# Neutrophils are immune cells preferentially targeted by retinoic acid in elderly subjects

**DOI:** 10.1186/1742-4933-7-10

**Published:** 2010-08-20

**Authors:** Régine Minet-Quinard, M Chantal Farges, Emilie Thivat, Cécile Deleine, Gilles Mayot, Julius Brtko, Josep Ribalta, Brigitte Winklhofer-Roob, Edmond Rock, M Paule Vasson

**Affiliations:** 1 Clermont University, Université d'Auvergne, EA4233, LB2MN, CRNH-A, BP10448, F-63000 Clermont-Ferrand, France; 2 Institute of Experimental Endocrinology, Slovak Academy of Sciences, Laboratory of Molecular Endocrinology, Vlarska 3, 833 06 Bratislava, Slovak Republic; 3 Universitat Rovira i Virgili, Unitat de Recerca de Lipids i Arteriosclerosi, Reus, Spain; 4 Karl-Franzens University, Institute of Molecular Biology, Biochemistry and Microbiology, Graz, Austria; 5 INRA, Unité des Maladies Métaboliques et des Micronutriments (UMMM), CRNH, F-63122 Saint-Genès-Champanelle, France; 6 Centre Jean Perrin, Service de Nutrition, F-63000 Clermont-Ferrand, France; 7 Human Nutrition & Metabolism Research and Training Center, Institute of Molecular Biosciences, Karl-Franzens University, Graz, Austria

## Abstract

**Background:**

The immune system gradually deteriorates with age and nutritional status is a major factor in immunosenescence. Of the many nutritional factors implicated in age-related immune dysfunction, vitamin A may be a good candidate, since vitamin A concentrations classically decrease during aging whereas it may possess important immunomodulatory properties via its active metabolites, the retinoic acids. This prompted us to investigate the immune response induced by retinoids in adults and elderly healthy subjects. Before and after oral supplementation with 13cis retinoic acid (0.5 mg/kg/day during 28 days), whole blood cells were phenotyped, and functions of peripheral blood mononuclear cells (PBMC) and polymorphonuclear cells (PMN) were investigated by flow cytometry and ELISA tests.

**Results:**

In both young adults (*n *= 20, 25 ± 4 years) and older subjects (*n *= 20, 65 ± 4 years), retinoic acid supplementation had no effect on the distribution of leukocyte subpopulations or on the functions of PBMC (Il-2 and sIl-2R production, membrane expression of CD25). Concerning PMN, retinoic acid induced an increase in both spontaneous migration and cell surface expression of CD11b in the two different age populations, whereas bactericidal activity and phagocytosis remained unchanged.

**Conclusions:**

We demonstrated that retinoic acid induces the same intensity of immune response between adult and older subjects, and more specifically affects PMN functions, i.e. adhesion and migration, than PBMC functions.

## Background

Efficacy of the immune system deteriorates with increasing age. This immunosenescence affects both the innate and the adaptive pathways immune pathways. The decline in the adaptive immune response has been well documented [1], highlighting an alteration in humoral immunity [2] and defective B and T-cell production [3]. Innate immunity is mediated by a diverse group of cell types and mechanisms, including monocytes/macrophages, natural killer (NK) cells, dendritic cells, neutrophils (PMNs), eosinophils and basophils, and by the elaboration of cytokines. Given that PMNs are the largest fraction of white blood cells and the first line of defense against pathogens, the literature contains numerous but inconsistent findings on the effect of aging on PMN numbers and functions (adhesion, chemotaxis, phagocytosis, radical oxygen species (ROS) production) (see review [4-7]) (table 1) [8-26]. These conflicting data may result from difficulties in distinguishing age-dependent specific changes in the immune system. Many other factors such as chronic disease, nutrition, and lifestyle have a profound effect on immunity, and could thus participate in more subtle age-related changes [27]. This immunosenescence is one reason older adults in general are vulnerable to infection and may play a central role in many degenerative diseases, yet the mechanisms are unclear. Consequently, understanding these immune dysfunctions is becoming increasingly important as the average life expectancies reach record levels.

**Table 1:** Effects of aging on neutrophil numbers and functions

	NO EFFECT	DECREASE
**NUMBER**	Chatta G. S., 1993, Gerontology Born J., 1995, Mech. Ageing Dev. Chatta G.S., 1996, Drugs Aging	De Martinis M., 2004, Immunol. Cell. Biol.

**ADHESION**	MacGregor R. R., 1990, J. Gerontol. Tortorella C, 1993, Mech. Ageing Dev. Esparza B., 1996, Immunol. Investig. Biasi D., 1996, Inflammation	Damtew B., 1990, Clin. Immunol. Immunopathol. De Martinis M., 2004, Immunol. Cell. Biol.

**CHEMOTAXIS**	Corberand J., 1981, J. Am. Geriatr. Soc Phair J. P., 1978, J. Infect. Dis. MacGregor R. R., 1990, J. Gerontol. Esparza B., 1996, Immunol. Invest.	Alonso-Fernandez P., 2008, J. Am. Geriatr. Soc. Niwa Y., 1989, Life Sci. Polignano A., 1994, Cytobios Wenisch C., 2000, J. Leukoc. Biol. Isgüt-Uysal V.N, 2003, Mech. Ageing Dev.

**PHAGOCYTOSIS**	Niwa Y., 1989, Life Sci. Chan S.S., 1998, Free Radic. Biol. Med.	Mege J.L., 1988. Neurobiol. Ageing Butcher S. K., 2001, J. Leukoc. Biol. Lord J.M. et al., 2001, Mech. Ageing Dev. Isgüt-Uysal V.N., 2003, Mech. Ageing Dev. Göcer P., 2005, Med. Princ. Prat.

**ROS PRODUCTION**		Wenisch C., 2000, J. Leukoc. Biol. Göcer P., 2005, Med. Princ. Pract.

Vitamin A (retinol) is known to play an essential role in the immune system and may be important for the optimal functioning of the innate and adaptive immune system [28]. Vitamin A deficiency is associated with exacerbated immunodeficiency [29], reduced or unbalanced lymphocyte counts [30], and deregulated antibody production [31]. Moreover, vitamin A supplementation reduces mortality from infectious diseases among children in areas where vitamin A deficiency is endemic [32]. These immunomodulatory properties have been demonstrated in both animal and humans studies [33,34], with somewhat contradictory results. The underlying cellular and molecular mechanisms governing the effect of vitamin A on the immune system remain elusive [35] but are primarily mediated via its acid derivatives, including all-trans-, 13-cis and 9-cis-retinoic acids [36]. The physiological effects of retinoids are mediated by members of two families of nuclear receptors: the RARα, RARβ and RARγ isotypes, and the RXRα, RXRβ and RXRγ isotypes. RARs bind both all-trans and 9-cis RA, whereas only 9-cis RA stereoisomers bind to RXRs.

Age-related alterations in vitamin A metabolism, particularly plasma retinol concentrations, have been reported in rats and humans [37,38] but also extend to retinoid receptors. Indeed, while retinoic acid receptor subtype expression in human PBMC follows the same distribution pattern in adult and old subjects, RXRβ expression is decreased in elderly subjects whereas RARα expression remains age-stable: RXRβ (39.8% in young men; 31.6% in elderly men) > RARγ (32.3% in young men; 33.4% in elderly men) > RXRα (17.8% in young men; 22.3% in elderly men) > RARα (10.3% in young men; 12.5% in elderly men) [39,40]

Nutritional status is a major factor in immunosenescence, and we have demonstrated that immunological adaptations to nutritional changes differ according to subject age [27]. Of the many nutritional factors implicated in age-related immune dysfunction, vitamin A may be a good candidate, firstly since vitamin A concentrations may be altered during aging, and secondly since vitamin A may possess important immunomodulatory properties. Reports on plasma vitamin A levels in free-living elderly populations are inconsistent, with some authors reporting a decrease whereas others describe no effect of aging on vitamin A [41-49]. These conflicting data may stem from the use of rather small or highly selective samples of older persons. However a recent study performed in a large sample of elderly subjects [50] confirmed that a non-negligible proportion of this population might be at risk of inadequate vitamin A status.

This prompted us to investigate the immune response induced by retinoids in adults and elderly healthy subjects. Since metabolism of vitamin A may be altered during aging, we investigated the biological actions of its active metabolite, i.e. retinoic acid, on immunocompetent cells. An oral 13-*cis*-retinoic acid supplementation was given to two groups aged 20-35 years and 60-75 years, and immune modulation was evaluated by phenotypic and functional assessment of blood leukocytes. In this cohort, we first measured retinoic acid receptor expression and demonstrated that RXRβ mRNA in PBMC was downregulated in elderly males but was upregulated after supplementation with 13-cis retinoic acid [40]. Thus, in light of this finding, this second part of our study (performed in the same cohort) was designed to investigate how these RXRβ expression modifications induced by retinoic acid translate biologically in terms of immune response in elderly subjects.

## Methods

### Subjects and treatment

Healthy male subjects (*n *= 40) were recruited from the Clermont-Ferrand area (France). Volunteers underwent a screening procedure that included medical history, physical examination, routine urine analysis and blood clinical chemistry profile. The study enrolled volunteers without malabsorption problems, liver disease, diabetes or hyperlipemia, who were not using steroids or other immunosensitive drugs, and who had not used vitamin supplements in the previous 30 days. The subjects were non-smokers and ingested alcohol no more than occasionally. The study was approved by the local ethical committee, and written informed consent was obtained from each subject before participation in the study. To assess the age effect, participants were picked from two age brackets: 20-35 years (mean: 25 ± 4 yrs) and 60-75 years (mean: 65 ± 4 yrs). The older subjects met medical criteria comparable to those specified by the SENIEUR protocol [51] for investigations into aging and the immune system. After examination by a dermatologist, the volunteers took 0.5 mg/kg b.w./day of 13-*cis*-retinoic acid (Curacné^®^, Pierre Fabre) for 28 days. All the volunteers were asked to avoid vitamin A-rich foods (mainly liver) and keep exposure to sunlight to a minimum, and were regularly contacted by the medical staff to note side effects.

### Venous blood sampling

Venous peripheral blood samples (10 ml) were collected into vacutainer tubes containing EDTA before (*T*_0_) and after the retinoid supplementation period (*T*_1_). Part (4 ml) of the total blood was used for the determination of ROS production by PMN cells and for blood leukocyte phenotyping. The rest (6 ml) was used to isolate both PMNs and PBMC for functional analysis.

At both time-points (*T*_0 _and *T*_1_), another venous peripheral blood sample (5 ml) was collected without anticoagulant, and centrifuged before isolation of sera. Sera were aliquoted and stored at -80°C for analysis of immune parameters.

### Plasma retinoic acid concentrations

13-*cis*-retinoic acid and retinol were assayed using a validated HPLC method as described by Wyss and Bucheli [52].

### Determination of serum immune parameters

Serum immunoglobulin (IgG, IgA, IgM) and complement fraction (C3, C4) concentrations were quantified by immunonephelemetry (array protein system, Beckman Coulter, Villepinte, France) using human antibodies (Beckman Coulter). Values are expressed in g/L. Serum soluble interleukin-2 receptor (sIl-2R) levels (expressed in pmol/L) were measured by ELISA (Immunotech kit, Beckman Coulter). The accuracy of our determinations is assured by a quality control scheme (VIGIL, Beckman Coulter, Villepinte).

### Count and phenotype of blood leukocytes

Lymphocyte subpopulations were determined by flow cytometry (Epics XL, Beckman Coulter) using a large panel of fluorochrome conjugated monoclonal antibodies (Beckman Coulter). A simple staining technique was used to identify mature T cells (CD3-PC5), B lymphocytes (CD19-ECD) and natural killer (NK) cells (CD56-PE). Dual-staining techniques were used to identify 'memory' (CD4-RD1/CD45RO-FITC) and 'naive' (CD4-RD1/CD45RA-FITC) populations of CD4+ cells. Triple-staining techniques were used to identify T-helper/inducer cells (CD3-PC5/CD4-RD1) and T-suppressor cells (CD3-PC5/CD8-ECD). To explore the expression of lymphocyte activation markers, whole blood cells were stained with anti-CD69 and anti-HLA-DR. In practice, peripheral venous blood samples were incubated with saturating concentrations of antibodies, and then depleted of red blood cells and fixed using the immunoprep reagent system (Beckman-Coulter). After addition of flow count spheres (Beckman Coulter), samples were finally analyzed by flow cytometry. Results are expressed in percentage of total lymphocyte population and in absolute values.

PMN receptors expression was determined by flow cytometry using fluorochrome-conjugated monoclonal antibodies (Beckman Coulter). The panel of monoclonal antibodies used was FITC anti-CD11b, FITC anti-CD18 and FITC anti-CD62L. Results are expressed in percentage of total PMN cell population and in absolute values. The fluorescence intensity of CD11b, expressed in arbitrary units (AU), is a qualitative reflection of the number of CD11b subset present on the PMN cell surface.

The results of our participation in the interlaboratory quality assurance programme (Immunotrol, Beckman Coulter) indicate the accuracy of the blood leukocyte phenotypes.

### Determination of blood leukocyte functions

Immune cells were isolated from whole blood on a discontinuous Ficoll-Hypaque density gradient (Histopaque 1077 and 1119, Sigma, Saint-Quentin-Fallavier, France). Isolated mononuclear cells and PMN were tested for purity (> 95%) and viability (> 95%) by flow cytometry.

In vitro *determination of Il-2 and its two receptors *(*sIl-2R and CD25)*: A mononuclear cell suspension (10^6 ^cells/ml) was incubated with and without phytohemagglutinin (5 μg/ml) (PHA from *Phaseolus vulgaris*, Sigma). After 48 h and 96 h of incubation, the cell-free supernatant of the culture medium was harvested and frozen at -80°C until analysis of Il-2 and its soluble receptor (sIl-2R) by ELISA tests (Beckman Coulter). Results are expressed in fmol/10^6^cells. For CD25 expression, cells were incubated with FITC antiCD25 monoclonal antibody (Il2rα chain) and flow count spheres (Beckman Coulter). Results are expressed as percentage of cells expressing CD25 and number of CD25 per cell.

#### PMN cell chemotaxis

PMN chemotaxis was determined by agarose assay (Indubiose A37, Biosepra, France) using formyl-methionyl-leucyl-phenylananine (fMLP, 10^-7 ^M) as chemotactic factor for 90 min (37°C, 5% CO_*2*_). Cell migration distances (in mm) were then measured microscopically in the direction of the chemoattractant (directed migration) and towards the control (spontaneous migration). Neutrophils chemotaxis index is expressed as the ratio of directed to spontaneous migration of PMN cells.

#### PMN hydrogen peroxide (H_2_O_2_) production

PMN H_2_O_2 _production was quantified by flow cytometry (Beckman Coulter) as described in [53]. Briefly, white blood cells (10^6 ^cells/ml) were stimulated with phorbol myristate acetate (PMA, 10^-8 ^M) or opsonized zymosan (10 mg/ml). H_2_O_2 _production was then evaluated by the fluorescence intensity of an intracellular oxidized probe (dihydrorhodamine 123). Results are expressed in AU.

#### Statistics

Results are expressed as means ± SEM. All statistical analyses were run on Statview SAS, version 5. The experimental design comprised two cross-fixed factors with the factor 'aging' as two classes (adults and elderly) and the factor 'nutritional treatment' as two classes (*T*_0_: basal state, *T*_1_: retinoic acid supplemented). This design allowed statistical analysis by two-way repeated-measures analysis of variance (ANOVA) in order to discriminate between the effects of aging (A), nutritional treatment (N), and their interaction (I). The level of significance was set at *p *< 0.05 for this test. When the ANOVA indicated significant interactions, the Newman-Keüls test was used to identify differences between individual means. When no significant interaction was found, the Newman-Keüls test was used to calculate and compare the marginal means.

## Results

### Clinical and biological features of the volunteers

Clinical examination showed that none of the 40 volunteers presented any severe side effects. Most of the volunteers had only noticed expected skin dryness. Retinoic acid supplementation and aging did not significantly affect cholesterolemia (adults *T*_0_: 5.28 ± 0.66 mM, *T*_1_: 5.61 ± 1.02 mM; elderly *T*_0_: 6.99 ± 1.09 mM, *T*_1_: 7.16 ± 1.73 mM) or triglyceridemia (adults *T*_0_: 1.02 ± 0.30 mM, *T*_1_: 1.11 ± 0.27 mM; elderly *T*_0_: 1.02 ± 0.25 mM, *T*_1_: 1.26 ± 0.49 mM).

### Plasma retinoic acid and retinol concentrations

Plasma 13-*cis*-retinoic acid concentrations were significantly affected by aging, supplementation and their interaction: they were increased at *T*_1 _in young adults and in elderly subjects and were significantly lower in older supplemented subjects than young adults (table 2). There was no difference in baseline plasma 13-*cis*-retinoic acid concentrations between young and older adults.

**Table 2:** Plasma retinoic acid and retinol concentrations at basal state (*T*_0_) and after supplementation with 13-*cis*-retinoic acid (*T*_1_)

	Young adults		Elderly		ANOVA
		
	***T***_**0**_	***T***_**1**_	***T***_**0**_	***T***_**1**_	
13-cis-retinoic acid (mg/l)	1.30 ± 0.08	202.61 ± 20	1.43 ± 0.07	149.95 ± 8.7	A, N, I
Retinol (μM)	2.76 ± 0.11	2.53 ± 0.09	3.14 ± 0.13	3.12 ± 0.15	A

X ± SEM; n = 20 subjects per group. Two-way repeated-measures ANOVA was performed to discriminate among the effects of age (A; p < 0.05), nutritional treatment (N; p < 0.05) and their interaction (I; p < 0.05).

For retinol, no significant interaction was found, and so comparison of marginal means was carried out, which indicated a significant effect of age (young: 2.64 ± 0.07 μM vs elderly: 3.13 ± 0.09 μM).

Plasma retinol concentrations were higher in the older subjects than the young adults but were not altered by retinoic acid supplementation (table 2).

### Serum immune parameters

Concentrations of IgA, IgG, IgM, C3, C4 and rIL2 s were not altered by age or by retinoic acid supplementation (IgG (g/L): adults *T*_0_: 12.30 ± 0.70, *T*_1_: 12.37 ± 0.60; elderly *T*_0_: 12.47 ± 0.65, *T*_1_: 12.57 ± 0.62; IgA (g/L): adults *T*_0_: 2.64 ± 0.30, *T*_1_: 2.25 ± 0.23; elderly *T*_0_: 3.09 ± 0.28, *T*_1_: 2.85 ± 0.28; IgM (g/L): adults *T*_0_: 1.47 ± 0.13, *T*_1_: 1.43 ± 0.14; elderly *T*_0_: 1.43 ± 0.13, *T*_1_: 1.60 ± 0.30; C3 (g/L): adults *T*_0_: 1.03 ± 0.07, *T*_1_: 1.09 ± 0.04; elderly *T*_0_: 1.14 ± 0.05, *T*_1_: 1.17 ± 0.05; C4 (g/L): adults *T*_0_: 0.25 ± 0.02, *T*_1_: 0.25 ± 0.02; elderly *T*_0_: 0.30 ± 0.02, *T*_1_: 0.31 ± 0.02; rIl2 s (fM): adults *T*_0_: 22 ± 3, *T*_1_: 23 ± 14; elderly *T*_0_: 24 ± 4, *T*_1_: 29 ± 7).

### White blood cell count

The leukocyte population, which includes lymphocytes, monocytes, and neutrophils was not altered by age or by retinoic acid supplementation (data not shown).

### Phenotypes and counts of blood leukocyte subpopulations

#### Lymphocytes

T lymphocytes expressed as a percentage or in absolute numbers were not modified by age or retinoic acid supplementation (Table 3). Age was associated with a lower absolute number of B lymphocytes without significant change in the proportion of this cell population. Retinoic acid supplementation induced no alteration in absolute number or percentage of B lymphocytes. NK lymphocytes were proportionally higher in elderly subjects than young adults but there was no effect of nutritional treatment. Absolute numbers of NK cells were not modified by aging or retinoic acid supplementation.

**Table 3:** Lymphocyte distribution at basal state (***T*_0_**) and after supplementation with 13-*cis*-retinoic acid (***T*_1_**)

	Young adults		Elderly		
			
	***T***_**0**_	***T***_**1**_	***T***_**0**_	***T***_**1**_	ANOVA
**T Lymphocytes**			±		
10^9^/L	1.56 ± 0.14	1.35 ± 0.09	0.99 ± 0.09	1.47 ± 0.45	-
%	68.1 ± 1.6	69.2 ± 1.3	64.7 ± 2.1	67.4 ± 1.7	-
					
**B Lymphocytes**					
10^9^/L	0.29 ± 0.04	0.26 ± 0.03	0.16 ± 0.02	0.22 ± 0.04	A
%	11.9 ± 0.9	12.6 ± 1.0	9.9 ± 0.8	11.2 ± 0.9	-
					
**NK Lymphocytes**					
10^9^/L	0.48 ± 0.06	0.33 ± 0.04	0.34 ± 0.04	0.38 ± 0.06	-
%	18.9 ± 1.8	15.8 ± 1.5	23.1 ± 22	21.0 ± 1.8	A

X ± SEM; n = 20 subjects per group. Lymphocytes are expressed both as percentages of the total lymphocyte number and as absolute values. Two-way repeated-measures ANOVA was performed to discriminate among the effects of age (A; p < 0.05), nutritional treatment (N; p < 0.05) and their interaction (I; p < 0.05).

As no significant interaction was found, comparison of marginal means was carried out, which indicated a significant effect of age on the absolute number (in 10^9^/L) of B lymphocytes (young: 0.276 ± 0.032 vs elderly: 0.186 ± 0.032) and on percentage of NK lymphocytes (young: 17.35 ± 1.69 vs elderly: 22.08 ± 1.97).

Neither the proportion (data not shown) nor absolute number(in 10^9^/L) of CD4^+ ^or CD8^+ ^cells was modified by aging or nutritional treatment (T4 lymphocytes: adults *T*_0_: 0.98 ± 0.09, *T*_1_: 0.87 ± 0.06; elderly *T*_0_: 0.61 ± 0.05, *T*_1_: 0.89 ± 0.20) (T8 lymphocytes: adults *T*_0_: 0.58 ± 0.05, *T*_1_: 0.48 ± 0.04; elderly *T*_0_: 0.38 ± 0.05, *T*_1_: 0.58 ± 0.20). Thus, the ratio of CD4^+ ^to CD8^+ ^cells was stable (adults *T*_0_: 1.78 ± 0.12, *T*_1_: 1.95 ± 0.14; elderly *T*_0_: 1.93 ± 0.24, *T*_1_: 2.11 ± 0.30).

Distribution between naive (CD4-CD45RA^+^) and memory (CD4-CD45RO^+^) T4 cells was modified by aging (table 4). Percentages (in %) and absolute values (in 10^9^/L) of CD4-CD45RA^+ ^cells were higher in young adults than elderly subjects. Percentage of CD4-CD45RO^+ ^cells was lower in young adults than older subjects. Retinoic acid supplementation induced no alteration in distribution between CD4-CD45RA^+ ^and CD4-CD45RO^+ ^cells (table 4).

**Table 4:** Naïve and memory T4 lymphocyte distribution at basal state (***T*_0_**) and after supplementation with 13-*cis*-retinoic acid (***T*_1_**)

	Young adults		Elderly		
			
	***T***_**0**_	***T***_**1**_	***T***_**0**_	***T***_**1**_	ANOVA
**CD4-CD45RO+**					
%	31.2 ± 1.74	29.36 ± 1.26	34.24 ± 1.81	35.88 ± 1.88	A
10^9^/L	0.751 ± 0.074	0.582 ± 0.052	0.527 ± 0.042	0.785 ± O.235	-
					
**CD4-CD45RA+**					
%	25.87 ± 1.70	24.32 ± 1.35	13.80 ± 1.92	15.18 ± 1.633	A
10^9^/L	0.620 ± 0.064	0.468 ± 0.222	0.219 ± 0.036	0.309 ± 0.078	A

X ± SEM; n = 20 subjects per group. Lymphocytes are expressed both as percentages of lymphocyte number and as absolute values. Two-way repeated-measures ANOVA was performed to discriminate among the effects of age (A; p < 0.05), nutritional treatment (N; p < 0.05) and their interaction (I; p < 0.05).

As no significant interaction was found, comparison of marginal means was carried out, which indicated a significant effect of age on the absolute number (in 10^9^/L) of CD4-CD45RA (young: 0.54 ± 0.04 vs elderly: 0.26 ± 0.04) and on percentage of CD4-CD45RA (young: 24.9 ± 1.1 vs elderly: 14.5 ± 1.3) and CD4-CD45RO (young: 30.5 ± 1.1: vs elderly: 35.1 ± 1.3).

Proportions of HLA-DR^+ ^cells or CD69^+ ^cells were not affected by aging or retinoic acid supplementation (HLA DR^+ ^cells: young adults, *T*_0_: 12.9 ± 0.9% *vs T*_1_: 14.3 ± 0.86%; elderly subjects, *T*_0_: 14.2 ± 0.8% *vs T*_1_: 16.3 ± 0.9%; CD69^+ ^cells: young adults: *T*_0_: 0.60 ± 0.06% *vs T*_1_: 0.49 ± 0.04%; elderly subjects: *T*_0_: 0.64 ± 0.11% *vs T*_1_: 0.71 ± 0.14%). The same pattern was found for the absolute counts of the two populations (data not shown).

#### PMN cells

Proportions of cells expressing CD11b, CD18, CD62L (in %) were not statistically different between young adults (99.9 ± 0.1, 99.8 ± 0.1, 84.1 ± 3.1 respectively) and older subjects (99.9 ± 0.1, 99.8 ± 0.2, 84.0 ± 3.1 respectively) and were not altered by retinoic acid supplementation (*T*_0_: 99.9 ± 0.1, 99.8 ± 0.1, 83.3 ± 2.9 *vs T*_1_: 99.9 ± 0.1, 99.9 ± 0.1, 83.8 ± 3.3, respectively). The same pattern was found for absolute numbers of cells expressing CD11b, CD18 and CD62 (data not shown). Concerning the fluorescence intensity of CD11b, since we found no significant interaction (p = 0.296), comparison of marginal means was carried out, which indicated a significant effect of nutritional treatment (*T*_0_: 10.3 ± 1.1 *vs T*_1_: 13.5 ± 1.2, p = 0.048) and no effect of aging (young adults: 13.4 ± 1.3 *vs *elderly subjects: 10.4 ± 0.9, p = 0.063).

### Blood leukocyte functions

#### Peripheral blood mononuclear cells

Il-2 production, sIl-2R production, and membrane expression of Il-2 receptor chain (% of CD25) were not significantly different between young adults and older subjects, and were not affected by retinoic acid supplementation (Table 5). Number of CD25 receptors per cell was lower in young adults than old subjects only in cells incubated for 96 h with PHA, and was not altered by retinoic acid supplementation (Table 5).

**Table 5:** Blood mononuclear cell functions at basal state (***T*_0_**) and after supplementation with 13-*cis*-retinoic acid (***T*_1_**)

Parameters	Incubation	Young adults		Elderly		
	Time	***T***_**0**_	***T***_**1**_	***T***_**0**_	***T***_**1**_	ANOVA
IL2 production	48 h	0.57 ± 0.24	0.75 ± 0.22	1.95 ± 0.75	0.77 ± 0.18	-

sIl-2R production	48 h	168 ± 24	186 ± 25	174 ± 27	201 ± 28	-

Membrane expression of CD25 (%)	48 h	27.5 ± 3.7	27.1 ± 2.8	21.3 ± 2.9	20.9 ± 2.4	-
	96 h	28 ± 2.9	34.4 ± 3.1	32.7 ± 3.9	32.4 ± 3.9	-

Number of CD25 receptors per cell	48 h	1214 ± 51	1366 ± 75	1157 ± 46	1262 ± 68	-
	96 h	874 ± 12	952 ± 22	1064 ± 67	1089 ± 27	A

X ± SEM; n = 20 subjects per group. Two-way repeated-measures ANOVA was performed to discriminate among the effects of age (A; p < 0.05), nutritional treatment (N; p < 0.05) and their interaction (I; p < 0.05).

Il-2 and sIl-2R productions were determined in culture media and expressed in fmol/10^6 ^cells

As no significant interaction was found, comparison of marginal means was carried out, which indicated a significant effect of age on number of CD25 receptors per cell (96 h) (young: 914.03 ± 14.16 vs elderly: 1077.24 ± 34.21)

#### Polymorphonuclear cells

**Figure 1:**
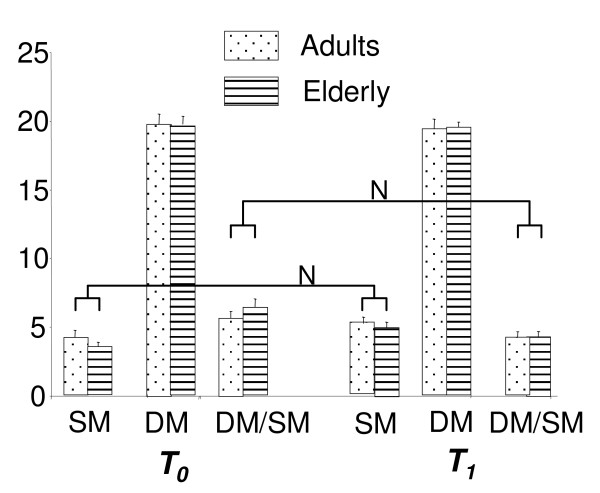
**Neutrophils chemotaxis**. Cell migration distances (in mm) are measured microscopically in the direction of the chemoattractant (directed migration (DM)) and towards the control (spontaneous migration (SM)). Neutrophils chemotaxis index is expressed as the ratio of directed to spontaneous migration of PMN cells. *x *± SEM; *n *= 20 subjects per group. Two-way repeated-measures ANOVA was performed to discriminate among the effects of age (A; *p *< 0.05), nutritional treatment (N; *p *< 0.05) and their interaction (I; *p *< 0.05). There was a significant effect of nutritional treatment for both chemotaxis index and spontaneous migration.

Chemotaxis index was not modified by aging but was decreased by retinoic acid supplementation due to an increase in spontaneous migration (Figure 1).

Hydrogen peroxide production (expressed in AU) by PMN suspension stimulated with PMA, a marker of bactericidal activity, was modified neither by aging (adults: 124 ± 10 *vs *elderly: 128 ± 13) nor by retinoic acid supplementation (*T*_0_: 119 ± 7 *vs T*_1_: 133 ± 15). The same result was obtained for phagocytosis, with H_2_O_2 _production (in AU) by PMN suspension stimulated with zymosan: young adults: 3.26 ± 0.16 *vs *elderly subjects: 3.23 ± 0.17; *T*_0 _3.42 ± 0.17 *vs T*_1 _3.03 ± 0.16.

## Discussion

An age-associated decline in immune function is well documented [54], yet the mechanisms involved are unclear, and it is becoming increasingly important to gain a deeper understanding of this dysfunction as average life expectancies reach record levels. Nutritional factors such as vitamin A may be implicated, since retinoids modulate different parts of the immune response components in both innate and adaptive immunity. While there is a significant body of research on the cellular and molecular mechanisms underlying these effects [55,56], most of these studies are either animal studies or *in vitro *studies on isolated immune cells. However, the complexity of nuclear retinoid receptors, their ligands and the various response elements in around a hundred genes makes it difficult to extrapolate results drawn from animal studies to *in vivo *human contexts. The present study brings original contributions, i) as it was performed in healthy humans and in both young and elderly groups, and ii) it investigates the immunomodulatory properties of retinoids directly through the active metabolite of vitamin A, which allow us to focus on alterations in vitamin A metabolism during aging. We have previously demonstrated a downregulated RXRβ expression in PBMCs isolated from healthy elderly subjects [39,40] and upregulated RXRβ expression after oral supplementation with 13-cis retinoic acid [40]. In order to better understand the role of retinoic acid in immunosenescence, we investigated the immune impact of these modified retinoic acid receptors by performing phenotype and functional assessment of blood leukocytes.

### Comparison between young adult and older healthy subjects

In our selected old population, alterations in immune capacity translated as a greater proportion of NK cells, a decrease in B lymphocytes and stable number of T-cells. A possible explanation for this result is a decline in T-cell turnover in the periphery of aged subjects with a decrease in T-cell generation and an increase in peripheral T-cell longevity [1]. In this T-cell population, we observed a decline in the proportion of naive T4 lymphocytes in parallel with an increase in memory T4 lymphocytes. This shift from a predominantly naive phenotype in the young to a predominantly memory phenotype in the aged subjects may explain the altered T-cell response widely reported in aged subjects.

This defect may be due not only to the high age of T-cells [57] but also to age-related differences in the lymphoid microenvironment [58], with a drop in concentration of IL7 cells which increase the lifespan of naïve CD4T cells and/or to intrinsic defects in aged T-cells [59,60].

### Effect of retinoic acid supplementation

Even if the dose of retinoic acid used was the same in the two populations studied, plasma retinoic acid concentrations were slightly lower in older subjects. This difference may not be explained by a difference in nutritional intake. Indeed, dietary intakes data did not show a decrease but an increase in α and β carotene intakes in older subjects compared with young adults. In addition, the bioavailability of these carotenoids with provitamin A activity is the same between young and older subjects [61]. This prompts us to suggest that retinoic acid metabolism is somewhat different between these two populations.

#### Lymphocyte phenotypes

In this *in vivo *human study, retinoic acid induced no modification in lymphocyte subsets in the two populations considered.

In adults, these results are consistent with the data of McKerrow et al. [62] but slightly different from those of Prabhala et al. and Watson et al. Employing *in vitro *[63] and *in vivo *[64] approaches, these authors showed that retinoic acid increased the percentage of both T-helper cells and NK cells and slightly decreased the percentage of T-suppressor cells.

In older subjects, Prabhala et al. [65] suggested that retinoic acid consumption may have a moderate immunoenhancing effect due to a significant increase in T4 and B cells. Fortes [66] reported similar results in an elderly population after 3 months of supplementation with vitamin A and zinc. The discrepancies between these reports may result from the duration of the study and the expression mode of the cell population.

As regards lymphocyte activation markers (CD25, CD69, HLA-DR), we report no effect of retinoic acid supplementation on these parameters in either young adult or old subjects.

#### Mononuclear cell functions

Il-2 production was not modified by retinoic acid supplementation in either young adult or older subjects. This result may be explained by a negative retinoic acid regulation on PHA-induced immune cell activation [67]. It is likely that retinoic acid reduces the protein production and gene expression of the Il-2 upregulated by PHA. This process is mediated through negative actions on c-fos and c-jun gene expression which would lead to the repression of the activity of AP-1, which interacts with the Il-2 promoter [67]. In addition, we observed a broad variability in Il-2 production which, as suggested by Engedal [55], may be explained by inter-blood donor variability in the strength of the anti-apoptotic effect of retinoic acid in T cells. The literature highlights major disparity in the effect of retinoic acid on Il-2 secretion, which may be explained by the differences in the immune system in the species studies. Retinoic acid inhibits *in vivo *interleukin-2 gene expression and T-cell activation in mice [68].

#### Serum immune parameters

Retinoic acid supplementation *in vivo *did not induce significant changes in immunoglobulin serum concentrations. The specific effect of *all-trans-*retinoic acid on immunoglobulin synthesis by B-lymphocytes has also been extensively reviewed by Semba et al. [33] and Ertesvag et al. [68]. They concluded that the conflicting results were probably attributable to differences in the experimental conditions and/or the origin of the immune cell. Some studies suggest that retinoic acid treatment of B cells could reduce immunoglobulin secretion, while others have demonstrated that retinoic acid may potentiate antibody-mediated immunity [32,69].

Retinoic acids may influence the balance between T-helper type 1 and T-helper type 2 response [70-73]. RXR signaling may play an important role in this Th-cell differentiation [74]. Indeed, RXR pathways enhance Th2 development in Ag-naive Th0 cells from DO11.10 TCR-transgenic mice [71]. Vitamin A enhances *in vitro *Th2 development *via *retinoid × receptor pathway whereas disruption of RXRα produced a Th1 bias *in vivo *[75]. Our finding that retinoic acid supplementation induced no increase in Il-2 production and no modification in immunoglobulin levels suggest no shift from Th1 to Th2 immune response in old subjects. Consequently, since we have previously shown that RXRβ expression is upregulated in old healthy subjects supplemented with 13-cis retinoic acid [40], these data may corroborate results from Iwata et al. [72] suggesting that RAR are also involved in the direct effect of RA on Th1/Th2 development.

#### PMN phenotypes and functions

PMN provide the first line of defense against infection. These cells play a pivotal role in the innate immune response and are the first to respond and target bacteria, fungi, protozoa and viruses. Briefly, the PMN cells adhere to the endothelium, chemotax towards the target organism or cell, phagocyte it and then use an arsenal of enzymes and active oxygen species to kill their target. Focusing on transendothelial migration, PMN rolling on activated endothelium and the adherence of PMN to the activated endothelium are critical steps. L-selectin (CD62l), which is a transmembrane glycoprotein, is an adhesion molecule on the PMN surface that promotes rolling adhesion. Firm adherence and diapedesis are mediated by β_2 _integrins, CD11b/CD18. Effect of retinoids on PMN functions has been well studied in animals. Functional studies performed in vitamin A-deficient animals show defects in chemotaxis, adhesion, phagocytosis, and reactive species production in neutrophils whereas vitamin A supplementation reverses these defects [76]. It has also been demonstrated that retinoids (retinol, retinoic acid, all-trans-retinal) stimulate superoxide production *in vitro *by guinea pig PMN [77-79], *in vitro *phagocytosis [80,81] bis dairy calves) and intracellular kill of live *Staphylococcus aureus *by blood PMN [82]. However, there is only limited research on the specific effect of vitamin A on neutrophil functions in humans, and the data produced came either from observation of malnourished subjects or from studies of vitamin A supplementation. In the first case, it is difficult to assess the specific effects of vitamin A since malnutrition is associated with multiple micronutrient deficiency. In the second case, the supplementation studies focused on specific groups, i.e. pregnant women and children, and the results are not consistent (for review, see [35]). In the present study performed on adult and elderly healthy subjects, we demonstrate that retinoids had a marked effect on two PMN functions, i.e. adhesion (increase in fluorescence intensity of a β2 integrin CD11b) and chemotaxis. The molecular pathways underpinning these effects have not yet been clearly established (for review, see [83]) but the Raf-1/MEK/Erk pathway [84,85] and intracellular adaptor molecules, i.e. PML-retinoic acid receptor alpha (RARα)-regulated adaptor molecule 1 (PRAM-1) [86] have been identified as essential for the integrin signaling cascade in neutrophils. Otherwise, b**2 **integrin (CD11b)-mediated adhesion of human polymorphonuclear neutrophils (PMN) has a functional impact on cytokine (Il8) production [87]. Therefore, our study in humans confirms some of the properties of retinoids on innate immunity previously demonstrated in animals, and contributes insight into the specific effect of retinoids on neutrophil functions during aging.

## Conclusions

We investigated the immunomodulatory properties of retinoic acid by employing a human *in vivo *approach rather than the experimental or animal study approaches reported to date. In addition, this is the first study performed in both young and old healthy populations to directly use the active metabolite of vitamin A, i.e. retinoic acid, and to measure plasma retinoid concentrations concomitantly with an evaluation of immune response. We elected to use the same dose of retinoic acid in the two populations, and we have demonstrated that immune response to retinoids was the same between adult and elderly subjects, despite lower plasma retinoic acid concentrations in the elderly subjects. Consequently, these results may suggest i) that retinoid metabolism evolves during aging, and ii) that sensitivity of immune cells is probably higher in old populations. To assess this second hypothesis, it would be interesting to run an *in vitro *experiment investigating the retinoid dose-response of immune cells isolated from adult and elderly donors. Among the immune cells studied, the effects of retinoids are more pronounced on PMN that PBMC. Indeed, while retinoic acid supplementation upregulates PBMC RXRβ expression in elderly subjects up to the levels found in young adults, this modification is without effect on PBMC phenotype and functions. Concerning PMN, we show that retinoic acid stimulates neutrophils adhesion and migration functions in the two aged populations *via *a process mediated by an increase in the β2 integrin CD11b expression. According to these results, retinoids may promote immune response in healthy adults and elderly by improving PMNs defense functions in terms of adhesion and chemotaxis. The nuclear pathway underpinning this effect remains to be explored. There is still limited data on the effects of retinoids on the immune system in humans during aging, and published results remain elusive. Our study adds a new facet to the understanding on this topic. However, further research is required to confirm the importance of retinoids in the immune system and especially in PMN functions in elderly subjects. In addition, considering that older subjects fail to elicit full immune response to viral and bacterial pathogens, and given the central role played by PMN in tissue injury during infection and the role of retinoids on PMN functions, research should be usefully directed towards investigating the effects of retinoic acid supplementation on immune response in elderly infected subjects.

## Competing interests

The authors declare that they have no competing interests.

## Authors' contributions

RMQ drafted the manuscript and performed the statistic tests. RMQ, MCF, ET, CD, GM performed the immunoassays and prepared the materials used in the experiment. RMQ, JB, JR, BWR, ER, MPV conceived of the study, participated in its design and coordination, contributed to the discussion of the results and the writing of the manuscript. All authors read and approved the final manuscript.
